# Analysis of lifestyle and metabolic predictors of visceral obesity with Bayesian Networks

**DOI:** 10.1186/1471-2105-11-487

**Published:** 2010-09-28

**Authors:** Alex Aussem, André Tchernof, Sérgio Rodrigues de Morais, Sophie Rome

**Affiliations:** 1 University of Lyon, F-69000, Lyon; University of Lyon 1, LIESP Laboratory, 69622 Villeurbanne, France; 2 Endocrinology and Genomics, Laval University Medical Center and Department of Nutrition, Laval University, Quebec, Canada; 3 RMND INSERM U870; INRA 1235, University of Lyon 1, 69622 Villeurbanne, France

## Abstract

**Background:**

The aim of this study was to provide a framework for the analysis of visceral obesity and its determinants in women, where complex inter-relationships are observed among lifestyle, nutritional and metabolic predictors. Thirty-four predictors related to lifestyle, adiposity, body fat distribution, blood lipids and adipocyte sizes have been considered as potential correlates of visceral obesity in women. To properly address the difficulties in managing such interactions given our limited sample of 150 women, bootstrapped Bayesian networks were constructed based on novel constraint-based learning methods that appeared recently in the statistical learning community. Statistical significance of edge strengths was evaluated and the less reliable edges were pruned to increase the network robustness. To allow accessible interpretation and integrate biological knowledge into the final network, several undirected edges were afterwards directed with physiological expertise according to relevant literature.

**Results:**

Extensive experiments on synthetic data sampled from a known Bayesian network show that the algorithm, called Recursive Hybrid Parents and Children (RHPC), outperforms state-of-the-art algorithms that appeared in the recent literature. Regarding biological plausibility, we found that the inference results obtained with the proposed method were in excellent agreement with biological knowledge. For example, these analyses indicated that visceral adipose tissue accumulation is strongly related to blood lipid alterations independent of overall obesity level.

**Conclusions:**

Bayesian Networks are a useful tool for investigating and summarizing evidence when complex relationships exist among predictors, in particular, as in the case of multifactorial conditions like visceral obesity, when there is a concurrent incidence for several variables, interacting in a complex manner. The source code and the data sets used for the empirical tests are available at http://www710.univ-lyon1.fr/~aaussem/Software.html.

## Background

### Introduction

Recently, Bayesian networks (BN) have become a very popular tool for biological network reconstruction [1-3], for genotype-to-phenotype relationship studies [4] and for clinical and microarray data aggregation [5,6]. BN are directed acyclic graphs (DAG) that model the probabilistic dependencies underlying the data. These graphical models are highly attractive for their ability to describe complex probabilistic interactions between variables. They offer a coherent and intuitive representation of uncertain domains of knowledge. The graphical part of BN reflects the structure of a problem, while local interactions among neighboring variables are quantified by conditional probability distributions. Learning a BN from data requires identifying both the model structure G and the corresponding set of model parameter values. Given a fixed structure, however, it is straightforward to estimate the parameter values. The task can be efficiently solved according to the maximum likelihood (ML) or maximum a posteriori (MAP) criterion under the assumption that the learning data contain no missing values [7,8]. As a result, research on the problem of learning BN from data is focused on methods for identifying the structure that best fits the data. Despite significant recent progress in algorithm development, the computational inference of network structure is currently still very much an open challenge in computational statistics [7,9]. To appreciate the complexity of learning a DAG, we note that the number of DAGs is super-exponential in the number of nodes [7].

Broadly speaking, there are two main approaches to BN structure learning. Both approaches have advantages and disadvantages. Score-and-search methods search over the space of structures (or the space of equivalence BN classes) employing a scoring function to guide the search. Another approach for learning BN structures, known as the constraint-based (CB) approach, follows more closely the definition of BN as encoders of conditional independence relationships. According to this approach, some judgments are made about the (conditional) dependencies that follow from the data and use them as constraints to construct a partially oriented DAG (PDAG for short) representative of a BN equivalence class. There are many excellent treatments of BN which surveys the learning methods [7,9]. When data sets are small, the relative benefits of the two approaches are still unclear. While none has been proven to be superior, considerable advances have been made in the past years in the design of highly scalable divide-and-conquer CB methods [10-14] in order to improve the network reconstruction accuracy when the number of samples is small.

In this study, we apply one of these CB algorithms, named Recursive Hybrid Parents and Children (RHPC), for representing the statistical dependencies between 34 clinical variables among 150 women with various degrees of obesity. Obesity is recognized as a disease in the U.S. and internationally by governments, health organizations, researchers and medical professionals. It is a complex multifactorial condition that needs to be studied by the means of multidisciplinary approaches involving biological expertise and new statistical and data mining tools. Features affecting obesity are of high current interest. Clinical data, such as patient history, lifestyle parameters and basic or even more elaborate laboratory analytes (e.g., adiposity, body fat distribution, blood lipid profile and adipocyte sizes) form a complex set of inter-related variables that may help better understand the pathophysiology of visceral obesity and provide guidance for its clinical management. Gregori et al. [15] performed a meta-analytic framework for the analysis of obesity and its determinants in children using Bayesian networks. Only seven lifestyle risk factors were considered as being potentially related to obesity in this population. To the best of our knowledge, our study is the first attempt to use BNs in the context of modeling the complex relationships between lifestyle and metabolic correlates of visceral obesity among women.

We use the bootstrapping method to generate more robust network structures as discussed in [6,16]. Statistical significance of edge strengths are evaluated using this approach. If an edge has a confidence above the threshold, it is included in the consensus network. Thus, if dependencies have enough support in the bootstrapping process they are captured and represented in the final consensus network. The confidence estimate assigned to each network edge is represented graphically on the final network. Such network represents a powerful computational tool for identifying putative causal interactions among variables from observational data. The consensus network graphically represents the possibly causal independence relationships that may exist in a very parsimonious manner [17]. In this study, special emphasis was placed on integrating physiological knowledge into the graph structure. Once the consensus PDAG was constructed from data, the remaining undirected edges were then directed according to our causal interpretation and additional latent variables were added to the graph for the sake of clarity, coherence and conciseness. The graphical representation provides a statistical profile of this sample of obese women, and meanwhile helps identifying the most important predictors of visceral obesity. Using the concept of a Markov Blanket we can identify all the variables that shield off the class variable from the influence of the remaining network. Therefore, BNs automatically perform feature selection by identifying the (in)dependency relationships with the class variable. We compare our findings with the results obtained using the same data and more traditional regression models.

### Bayesian networks

Formally, a BN is a tuple <G, *P *> where G = <**U**, **E **> is a directed acyclic graph (DAG) with nodes representing the variables in the domain **U**, and edges representing direct probabilistic dependencies between them. *P *denotes the joint probability distribution on **U**. The BN structure encodes a set of conditional independence assumptions: that each node *X_i _*is conditionally independent of all of its nondescendants in G given its parents $Pa_{i}^{G}$. These independence assumptions, in turn, imply many other conditional independence statements, which can be extracted from the network using a simple graphical criterion called d-separation [8].

We denote by *X *⊥_*P *_*Y*|**Z **the conditional independence between *X *and *Y *given the set of variables **Z **where *P *is the underlying probability distribution. Note that an exhaustive search of **Z **such that *X *⊥_*P *_*Y*|**Z **is a combinatorial problem and can be intractable for high dimension data sets. We use $X{⊥}_{G}Y|Z$ to denote the assertion that *X *is d-separated from *Y *given **Z **in G. We denote by **dSep**(*X*, *Y*), a set that d-separates *X *from *Y*. If <G, *P *> is a BN, *X *⊥_*P *_*Y*|**Z **if $X{⊥}_{G}Y|Z$. The converse does not necessarily hold. We say that <G, *P *> satisfies the *faithfulness condition *if the d-separations in G identify *all and only *the conditional independencies in *P*, i.e., *X *⊥_*P *_*Y*|**Z **if and only if (iff) $X{⊥}_{G}Y|Z$. Two graphs are said *equivalent *iff they encode the same set of conditional independencies via the d-separation criterion. The equivalence class of a DAG G is a set of DAGs that are equivalent to G. [8] established that two DAGs are equivalent iff they have the same underlying undirected graph and the same set of v-structures (i.e., uncoupled head-to-head meetings *X *→ *Y *← *Z*). So we define an *essential *graph (also called a DAG pattern) for a Markov equivalence class to be the partially directed acyclic graph (PDAG), that has the same links as the DAGs in the equivalence class and has oriented all and only the edges common to all of the DAGs in the equivalence class. The directed links in the essential graph are called the *compelled *edges [7].

An important concept of BN is the Markov blanket of a variable, which is the set of variables that completely shields off this variable from the others. In other words, a Markov blanket **M***_T _*of *T *is any set of variables such that *T *is conditionally independent of all the remaining variables given **M***_T _*. A Markov boundary, **MB***_T _*, of *T *is any Markov blanket such that none of its proper subsets is a Markov blanket of *T*. Suppose <G, *P *> satisfies the faithfulness condition. Then, for all *X*, the set of parents, children of *X*, and parents of children of *X *is the unique Markov boundary of *X*. A proof can be found for instance in [7]. We denote by $PC_{T}^{G}$, the set of parents and children of *T *in G, and by $SP_{T}^{G}$, the set of *spouses *of *T *in G, i.e., the variables that have common children with *T*. These sets are unique for all G, such that <G, *P *> satisfies the faithfulness condition and so we will drop the superscript G.

### Bayesian network structure learning

Automatically learning the graph structure of a BN is a challenging topic of pattern recognition that has attracted much attention over the last few years. CB methods systematically check the data for conditional independence relationships and try to construct a partially directed graphical structure (also called a perfect map) that encodes perfectly the set of independencies. Typically, these algorithms run a *χ*^2 ^independence test when the dataset is discrete and a Fisher's *z *test when it is continuous in order to decide on dependence or independence, that is, upon the rejection or acceptance of the null hypothesis of conditional independence. Therefore, conditional independencies that are read off from the BN structure are in total agreement with the conditional independencies that are obtained by the statistical tests. Very powerful, correct, scalable and data-efficient CB algorithms have been recently proposed [10-12]. They are correct (or sound) in the sense that they return the correct essential graph under the assumptions that the independence tests are reliable and that the learning database is a sample from a distribution *P *faithful to a DAG G. The (ideal) assumption that the independence tests are reliable means that they decide (in)dependence iff the (in)dependence holds in *P*. In this paper we adopt one of these CB approaches [11,18]. The essential graph is obtained by running an algorithm called *Recursive HPC *(RHPC), where HPC stands for Hybrid Parents and Children.

## Results

### Simulation experiments on artificial data

As RHPC relies on HPC to build the whole network structure, we conducted several experiments on synthetic data to assess the comparative performance of HPC, and two algorithm proposals that appeared recently in the literature, namely MMPC [12] and GetPC [10]. The source code (C++) of HPC as well as all data sets used for the empirical tests are available at *http://www710.univ-lyon1.fr/~aaussem/Software.html*. The authors' implementation of MMPC and GetPC can be found respectively at http://discover.mc.vanderbilt.edu/discover/public and http://www.ida.liu.se/~jospe. MMPC was deemed one of the best CB algorithms in [12] and GetPC was used recently in [2] for modeling gene networks. We also report the performance of our weak learner Inter-IAPC for comparison. For GetPC and MMPC, we used the softwares proposed by the respective authors (see footnote). The confidence threshold of the independence test was fixed to α = 0.05 for all algorithms. All the data sets used for the empirical experiments presented in this section were sampled from a bio-realistic network that has been previously used as benchmark for BN learning algorithms, namely *Insulin *(35 nodes and 52 edges). The Insulin network [19] was chosen purposely as it consists of the same number of nodes as our dataset. Four sample sizes have been considered: 200, 500, 1000 and 2000. For each sample size, 100 data sets were sampled. We do not claim that this benchmark resembles our real-world problem, however, it makes it possible to compare the outputs of the algorithms.

**Figure 1:**
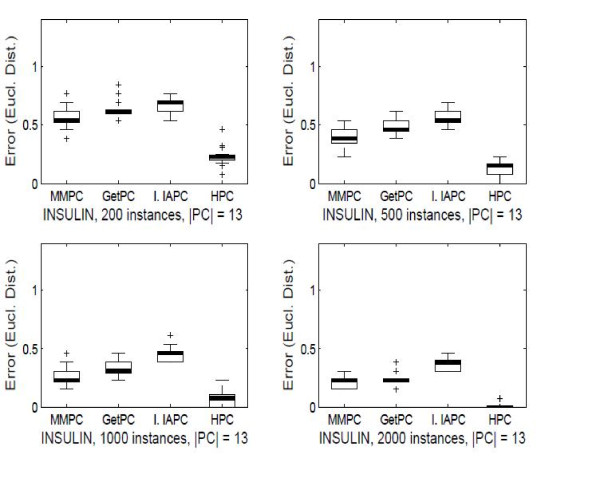
**Validation of the learning method on the Insulin benchmark**. Empirical experiments on synthetic data sets from the *Insulin *BN. Each algorithm is run on the node having the largest neighborhood (13 nodes). Four sample sizes were considered: 200, 500, 1000 and 2000. The figure shows the distribution over 100 data sets of the Euclidean distance from perfect precision and recall, in the form of boxplots.

All four algorithms were run on the target node having the largest degree (13 neighbors) in the Insulin BN to increase the difficulty of the task. The variables in the output of the algorithms were compared against the true neighbors. To evaluate the accuracy, we combined precision (i.e., the number of true positives in the output divided by the number of nodes in the output) and recall (i.e., the number of true positives divided by 13, the size of the true PC set) as $\sqrt{{\left(1-precision\right)}^{2}+{\left(1-recall\right)}^{2}}$, to measure the Euclidean distance from perfect precision and recall, as proposed in [10]. Figure 1 summarizes the variability of the Euclidean distance over 50 data sets in the form of quadruplets of boxplots, one for each algorithm (i.e., MMPC, GetPC, Inter-IAPC and HPC). The advantage of *HPC *against the other three algorithms is clearly noticeable. HPC outperforms the other algorithms in terms of Euclidean distance from perfect precision and recall.

### Simulation experiments on the sample of women

**Figure 2:**
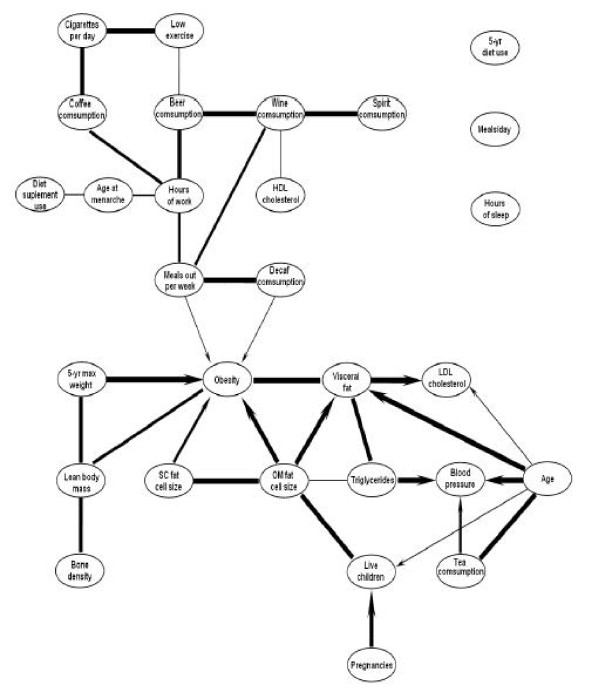
**Consensus PDAG of visceral obesity related variables in women returned by RHPC**. Consensus PDAG obtained by running RHPC on bootstrapped samples. Labels are self-explanatory. Line thickness corresponds to the relative edge strength.

The consensus PDAG obtained by running RHPC on the present sample of women is shown in Figure 2. Line thickness corresponds to the relative confidence of the edges. The edges that appeared more than 25% in the networks were included in the aggregate PDAG. The threshold was tuned on the previous Insulin benchmark samples to maximize accuracy. As may be seen, the directionality of the arrows was partially identifiable: 14 edges out of 34 were directed, indicating the presence of several robust uncoupled head-to-head meetings (*T *→ *Y *← *X*).

#### Physiological knowledge integration into the model

**Figure 3:**
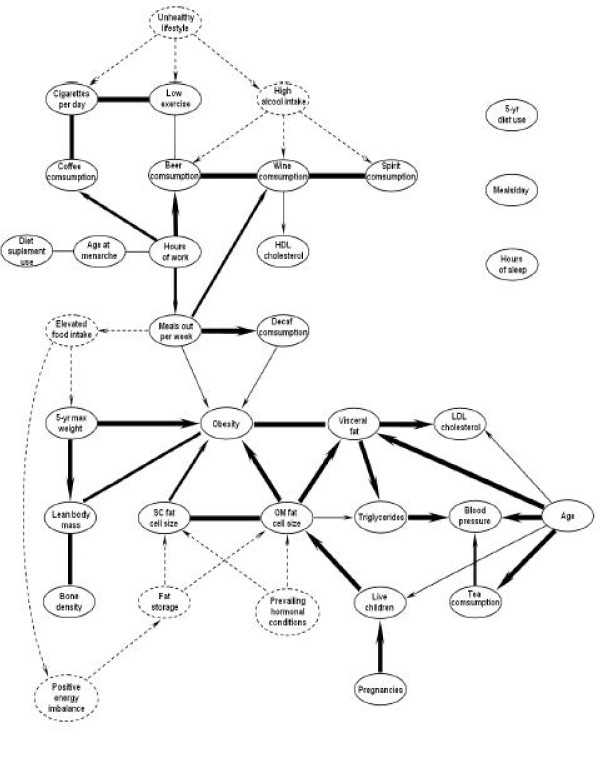
**BN of visceral obesity related variables in women after physiological knowledge integration into the graph**. PDAG of Figure 2 oriented according to biological knowledge. Dash nodes and arrows are latent variables that were added based on current literature.

Several interconnected groups of variables were identified, e.g., beer consumption, wine consumption and spirit consumption; cigarettes per day and low exercise; OM and SC fat cell sizes. In each of these densely connected subgraphs, the variables were highly interdependent and a common cause is likely to explain the observed correlations. Hence, we added some extra nodes and directed some of the links according to physiological knowledge available in the literature. The result is the partially directed acyclic graph (PDAG) that is shown in Figure 3. Dashed nodes and arrows are the latent variables that were added for sake of clarity and coherence. By definition, these latent variables are not observed, nor recorded in our data set. For example, the variable high alcohol intake was added as a common "cause" to beer consumption, wine consumption and spirit consumption; the variable unhealthy lifestyle was added as a common cause to cigarettes per day, high alcohol intake and low exercise; the latent variables fat storage and prevailing hormonal conditions were added as two distinct common causes to SC fat cell size and OM fat cell size.

Almost all the undirected edges were oriented based on current literature as follows. Edges directed from the age variable were oriented based on the well-documented impact of ageing on visceral adipose tissue accumulation, blood pressure and plasma LDL-cholesterol levels [20,21]. The edge between age and tea consumption is based on the 2004 Canadian Community Health Survey, which showed a steady increase in tea consumption from 19 to more than 71 years of age [22]. The edge between tea consumption and blood pressure was oriented based on literature showing lower cardiovascular disease risk in tea consumers [23] and a direct effect of black tea consumption on peripheral blood flow and arterial stiffness [24]. The edge between age and the number of live children was attributed to the slight decrease in Canadian birth rates observed between 1961-66 and 1981-86 [25], which corresponds approximately to the period in which women of the study had their children. Accordingly, older women of the sample were more likely to have delivered slightly more children. Orientation of the edge between the number of pregnancies and the number of live children is self-explanatory.

The edge between the number of live children and OM fat cell size was derived from literature supporting that post-pregnancy weight retention is an important risk factor for obesity [26]. The finding of a specific association between the number of children and OM fat cell size was novel and warrants further investigation. The edges between OM and SC fat cell sizes and the variables obesity or visceral fat is self explanatory since the excess adipose tissue mass of obese or abdominal obese individuals is constituted of larger fat cells. Associations between fat cell size and obesity have been previously observed [27]. The edges between visceral fat or large OM fat cells and metabolic variables such as LDL-cholesterol, triglycerides and blood pressure was oriented based on the 'portal vein hypothesis', which states that visceral fat is a causal agent for metabolic disturbances [28]. However, this hypothesis has not yet been fully proven as operative and has been challenged by a number of investigators. Further studies are required to firmly establish causality. However, the fact that the association between visceral fat and metabolic disturbances is independent from overall obesity is well-accepted [29,30]. The edges between the various components of body composition (i.e., bone density, lean body mass and obesity) were logical but it was difficult to provide causal direction between these variables. Indeed, many genetic, epigenetic, developmental and environmental factors can contribute to determine body built of a given individual. Moreover, the sizes of all compartments generally evolve in a more or less coordinated manner throughout the individual's existence [31,32]. It was expected that the variable 5-yr maximal weight would be a strong correlate of the level of obesity and lean body mass since these variables are the main components of body composition [32] and that most patients reported a stable weight in the five years preceding their inclusion in the study.

The edges around the number of hours of work and the number of meals out per week were oriented based on the demonstration that increased working time was associated with food choice coping strategies [33], which we suggest is reflected by the edges to number of meals out per week, beer, wine and coffee consumption. On the other hand, the number of meals out per week was related to obesity. Accordingly, the frequency of restaurant food consumption was previously found to be positively related to body fatness [34]. Wine consumption was related directly with plasma levels of HDL-cholesterol. This edge was oriented based on epidemiological data showing a protective effect of moderate wine consumption on HDL-cholesterol levels [35]. Low leisure time physical activity was linked together with smoking habits under a latent causal variable that we termed unhealthy lifestyle. These variables were also linked with coffee and beer consumption, but had no direct link with the level of obesity. We were unable to provide orientation for these edges. Moreover, we were not able to readily explain a small number of edges. For example, the link between age at menarche, which reflects timing of puberty, and dietary supplement use is not intuitive. Further analyses and other samples will be required to clarify this apparent association.

#### Statistical validation

We noticed from the PDAG that OM fat cell size, visceral fat, blood pressure, tea consuption and age belonged to the triglycerides Markov boundary, though the edge between OM fat cell size and triglycerides was only moderate in strength. The influence of OM fat cell size on triglycerides was mostly mediated by visceral fat. We observed that age and triglycerides were marginally independent according to the d-separation rule. However, they became dependent conditioned on visceral fat. The PDAG was consistent with multivariate linear regression analyzes performed a posteriori on the sample (Table 1). In model 1, plasma triglyceride levels were predicted using computed tomography-measured visceral adipose tissue area (visceral fat variable) and total body fat mass (which is included in the variable obesity). Visceral fat explained 31.9% of the variance in triglyceride levels whereas overall obesity was not a significant predictor of triglyceride levels. A similar analysis in which plasma triglyceride levels were predicted by OM and SC fat cell size was also performed (Table 1, model 2). OM fat cell size explained 21.2% of the variance in triglyceride levels, whereas SC fat cell size was not a significant predictor of triglyceride levels in the model.

**Table 1:** Prediction of plasma triglyceride levels

	Independent variable	Parameter estimate	P value	**Partial *R***^**2**^**× 100**	**Total *R***^**2**^**× 100**
Model l	Visceral fat	1.0568	0.0001	31.9	31.9
	Obesity (Total body fat mass)	0.0425	NS	0.0	

Model 2	OM fat cell size	0.0088	0.0001	21.2	21.9
	SC fat cell size	0.0031	NS	0.0	

Multivariate regression models for the prediction of plasma triglyceride levels with adiposity measures (top); or fat cell size in the omental (OM) and subcutaneous (SC) compartment (bottom). Variables with non-normal distributions (Shapiro-Wilk test p < 0.05) were log-10- or Box Cox-transformed for the analysis.

## Discussion

The purpose of this paper was to introduce the BN methodology in the context of clinical studies, specifically obesity, and to show its effectiveness, as a component of general data mining/knowledge discovery approaches in epidemiology research. We have evaluated a consensus BN learning approach based on boot-strapping techniques on synthetic data with satisfactory results. Although our approach did not use any prior information, it was successful in uncovering biologically relevant dependencies and conditional independencies. Once the most interesting dependencies are ascertained, traditional statistical methods (e.g. linear or logistic regression, etc.) can be used to rigorously scrutinize the resulting smaller subnetworks.

In this study, special emphasis was put on integrating physiological expertise and statistical data analysis together. It is well beyond the scope and purpose of this paper to delve deeper into the problem of inferring causalities from observational data. However, the usefulness of BN stems partly from their causal interpretation. As we have seen, the graphical representation is useful as it allows tighter collaboration between the modeler and the biologist. The integration of medical knowledge into data-driven models is not only desirable, but it is also far easier and less subjective than constructing the whole BN with *a priori *knowledge. In this spirit, most edges were directed according to plausible causal inference although interpretation of edges as carriers of information does not necessarily imply causation.

## Conclusions

Thirty-four predictors related to lifestyle, adiposity, body fat distribution, blood lipids and adipocyte sizes have been considered as potential correlates of visceral obesity in women. The analysis was performed with a novel scalable and effective constraint-based bayesian network structure learning algorithm called RHPC.

From a biological point of view, the present study confirms, among other interesting findings, that visceral fat is the predominant predictor of triglyceride levels in obese individuals. It is reassuring that an unsupervised BN analysis uncovered previously established relationships between visceral fat, blood pressure, aging and triglyceride levels. The advantage of BN method is not that it will identify the "true causes", but rather that it will perform initial data exploration to unearth new knowledge in a semi-automated and rapid fashion.

In conclusion, we suggest that BNs are valuable data mining tools for the analysis of clinical data. In addition, BNs can explicitly combine both expert knowledge from the field and information studied from the data. A need for such multi-step processes (hypothesis generation step followed by a traditional hypothesis testing step) is essential. Finally, an extension to our existing framework would be to consider Bayesian model averaging as an alternative to a single consensus model selection. This extension is currently underway.

## Methods

### The Recursive Hybrid Parents and Children algorithm

RHPC is based on the faithfulness assumption. As RHPC calls HPC on each node, we start discussing HPC first. HPC receives a node *X *and returns its adjacent nodes **PC***_X_*. Under this faithfulness assumption, *X *and *Y *are not adjacent in G if and only if ∃ **Z **∈ **U**\{*X*, *Y*} such that *X *⊥ *Y*|**Z **[7]. As an exhaustive search of **Z **is intractable for high dimension data sets. HPC perfoms a heuristic search with a severe restriction on the maximum conditioning size in order to significantly increase the reliability of the statistical independence tests. Note that other similar 'Parent and Children' learning procedures were proposed recently in the machine learning literature, namely MMPC [12] and GetPC [10]. They could be used as well. Nonetheless HPC was favored in a recent evaluation using the same conditional independence test, over a range of different networks, sample sizes and number of variables [11].

Formally, HPC can be viewed as an ensemble method for combining many weak PC learners in an attempt to produce a stronger PC learner. The algorithm was designed in order to endow the search procedure with the ability to: 1) handle efficiently data sets with thousands of variables but comparably few instances; 2) deal with datasets which present some deterministic relationships among the variables; 3) be correct under the faithfulness condition; and 4) be able to learn large neighborhoods. HPC is based on three subroutines: *Data-Efficient Parents and Children Superset *(DE-PCS), *Data-Efficient Spouses Superset *(DE-SPS), and *Interleaved Incremental Association Parents and Children *(Inter-IAPC), a weak PC learner based on Inter-IAMB [36] that requires little computation. HPC was shown to be correct in the sample limit under the faithfulness assumption [11,18]. For the sake of conciseness, we only discuss the main HPC routine. The algorithm details are omitted here for brevity: RHPC and its sub-routines are thoroughly described in additional file 1 for the sake of conciseness.

HPC may be thought of as a way to compensate for the large number of false negative nodes, at the output of the weak PC learner with few data cases, by performing extra computations. HPC receives a target node *T*, a data set D and a set of variables **U **as input and returns an estimation of **PC***_T_*. It is hybrid in that it combines the benefits of incremental and divide-and-conquer methods. The procedure starts by extracting a superset **PCS***_T _*of **PC***_T _*(line 1) and a superset **SPS***_T _*of **SP***_T _*(line 2) with a severe restriction on the maximum conditioning size (**Z *** <*= 2) in order to significantly increase the reliability of the tests. A first candidate PC set is then obtained by running the weak PC learner on **PCS***_T _*∪ **SPS***_T _*(line 3). The key idea is the decentralized search at lines 4-8 that includes, in the candidate PC set, all variables in the superset **PCS***_T _*∪ **SPS***_T _*that have *T *in their vicinity. Note that, in theory, *X *is in the output of Inter-IAPC(*Y*) if and only if *Y *is in the output of Inter-IAPC(*X*). However, in practice, this may not always be true, due to the statistical test errors that should appear, especially with few data samples. The decentralized search enables the algorithm to handle large neighborhoods while still being correct under faithfulness condition.

The essential graph is obtained by running HPC on the every node and by directing the *compelled *edges as shown in RHPC. Note that HPC must have found *dSep*(*X*, *Y *) (at line 5 of RHPC) and have cached it for later retrieval. Alternatively, HPC can be run recursively on the adjacent nodes of a target variable in order to establish a local graph without having to construct the whole BN first as discussed in [2]. RHPC applies standard techniques at lines 4-19 to identify the compelled edges. The reader is directed to [7], pp. 538, for further details. The *correctness *and *completeness *of the edge orientation in RHPC are demonstrated in [37].

### Network aggregation

As discussed in the introduction, our practical goal is to extract a BN structure that encodes the conditional independencies between 34 variables given our sample of 150 women. The most common approach to discovering the structure is to use learning with model selection to provide us with a single model. However, model selection is known to be sensitive to the particular data set, especially with few instances. Had we sampled another data set of the same size from the same distribution, model selection would have learned a different model [16]. So we cannot simply accept our chosen structure as a true representation of the under-lying distribution. Averaging over the sampled structures that are generated by a sampling process produces models that are more robust, have greater confidence and place less reliance on a single dataset. Several approaches exist: generating samples of the BN structure from its marginal posterior distribution using Monte Carlo Markov chain (MCMC) [16,38-40], using bootstrapping methods for computing a statistical confidence features within a BN [6,16]. In this study, we make use of the bootstrapping method to generate a more robust network structure. The 're-shuffled' dataset is generated from the original dataset (re-sampling with replacement), the graph is built from this re-shuffled set and then the procedure is repeated a sufficient number of times. Confidence in a particular edge is measured as a percentage of the number of times this edge actually appears in the set of reconstructed graphs. If an edge has a confidence above the threshold, it is included in the consensus network. Thus, if dependencies have enough support in the bootstrapping process, they are captured and represented in the final consensus network. When computing confidence estimates, we define a feature as the existence of an edge between two nodes in the PDAG. Thus, the bootstrapped network has a confidence estimate assigned to each network edge. Where directed edges are present in a PDAG, they contribute only to the confidence estimate for the edge in that direction, whereas undirected edges contribute to the confidence estimate for an edge in both directions. If an edge has a confidence above the threshold, it is included in the consensus PDAG, and if edges are found in both directions (e.g. from node *X_i _*→ *X_j _*and *X_j _*← *X_i_*), then the edge is undirected. Thus, if directional dependencies have enough support in the bootstrapping process, they will be captured and represented in the final PDAG.

### Biological data

The sample of 150 obese women used for these analyzes consists of 34 variables related to lifestyle such as alcohol consumption, smoking habits, leisure time activity and eating patterns. Dual energy x-ray absorptiometry was used to obtain whole-body measures of body composition (bone density, lean body mass, total body fat mass). Computed tomography was used to assess body fat distribution at the abdominal level. These measures include adipose tissue areas of the abdominal fat compartments located subcutaneously and inside the abdominal cavity (visceral fat). Finally, the variables examined also include average adipocyte sizes measured both in the omental (OM) and subcutaneous (SC) adipose tissue compartments from adipose tissue samples obtained during surgery. Women included in these analyses have been the object of previous publications on other topics [41,42]. All women who participated in the protocols signed an informed consent document. The projects were approved by the ethics committee of Laval University Medical Center.

## Authors' contributions

SRM and AA designed and implemented the learning algorithms, SRM and AT performed the tests, AT and SR analyzed the results. AA chose the mathematical framework and supervised the work. AA and AT wrote the manuscript. SR and SRM critically reviewed the manuscript. All authors read and approved the final manuscript.

## Supplementary Material

Additional file 1 **Description of the Recursive Hybrid Parents and Children algorithm**. This file contains a detailed discussion of our algorithm called Recursive Hybrid Parents and Children (RHPC). RHPC takes a data set as input and returns a partially oriented DAG (PDAG for short) representative of a bayesian network equivalence class. The latter is obtained by directing the *compelled *edges of the skeleton. The skeleton is obtained by running an algorithm called Hybrid Parents and Children (HPC) algorithm recursively on every node. RHPC is shown to be sound in the sample limit. Click here for file
