# Supplementary material for: Analysis of lifestyle and metabolic predictors of visceral obesity with Bayesian Networks
Source: BMC Bioinformatics. 2010 Sep 28;11:487. doi: 10.1186/1471-2105-11-487 (PMC2955704; doi:10.1186/1471-2105-11-487)
Supplement: Additional file 1 — Description of the Recursive Hybrid Parents and Children algorithm. This file contains a detailed discussion of our algorithm called Recursive Hybrid Parents and Children (RHPC). RHPC takes a data set as input and returns a partially oriented DAG (PDAG for short) representative of a bayesian network equivalence class. The latter is obtained by directing the compelled edges of the skeleton. The skeleton is obtained by running an algorithm called Hybrid Parents and Children (HPC) algorithm recursively on every node. RHPC is shown to be sound in the sample limit. [file 1471-2105-11-487-S1.PDF]

Description of the Recursive Hybrid Parents and Children algorithm

Alex Aussem André Tchernof
Sérgio Rodrigues de Morais Sophie Rome

July 9, 2010

Abstract

We discuss, in more details, our algorithm called Recursive Hybrid Parents and Children (RHPC). RHPC takes a data set as input and returns a partially oriented DAG (PDAG for short) representative of a bayesian network equivalence class. The latter is obtained by directing the *compelled* edges of the skeleton. The skeleton is obtained by running an algorithm called Hybrid Parents and Children (HPC) algorithm recursively on every node. RHPC is shown to be sound in the sample limit.

1 Introduction

Constraint-based (CB) bayesian network (BN) structure learning methods are plagued by a severe problem: the number of false negatives (missing variables) increases swiftly as the size of the PC set increases. There are mainly two explanations for this phenomenon. The first is the unreliability of the conditional independence tests as the conditioning sets become large. Large conditioning sets produce sparse contingency tables and unreliable tests. This is why it is difficult to learn the neighborhood of a node having a large degree with CB methods. The number of possible configurations of the variables grows exponentially with the size of the conditioning set. This well known problem is common to all CB methods and has led several authors to reduce, as much as possible, the size of the conditioning sets with a view to enhancing the *data-efficiency* of their methods [3]. The second reason is that the decisions for a link to be created between two nodes in the final graph are often too severe and conservative. In practice, these problems plague all CB methods when variables have many adjacent nodes and relatively few instances. Moreover, CB procedures suffer from another difficulty: they fail to reconstruct correctly the skeleton when some approximate deterministic relationships (ADR) exist among groups of variables. ADRs are pitfalls to watch out for when a conservative procedure is run on data because it causes the method to miss weakly associated pairs of variables [8].

In this supplementary document, we discuss in more detail the algorithm called Recursive Hybrid Parents and Children (RHPC) that was used in our experiments and we briefly explain how it alleviates the problems discussed above. RHPC takes a data set as input and returns a PDAG representative of a bayesian network equivalence class (called an essential graph). The latter is obtained by directing the *compelled* edges of the skeleton. The skeleton is

obtained by running an algorithm called Hybrid Parents and Children (HPC) recursively on each node [5–7].

2 The Recursive Hybrid Parents and Children algorithm

The essential graph is obtained by running RHPC (Algorithm 1), based on the faithfulness assumption. As RHPC calls HPC (Algorithm 2) on each node, we start discussing HPC first. HPC receives a node X and returns its adjacent nodes $\mathbf{PC}_X$. Under this faithfulness assumption, X and Y are not adjacent in $\mathcal{G}$ if and only if $\exists \mathbf{Z} \in \mathbf{U} \setminus \{X \cup Y\}$ such that $X \perp Y | \mathbf{Z}$ [2]. As an exhaustive search of $\mathbf{Z}$ is intractable for high dimension data sets. HPC performs a heuristic search with a severe restriction on the maximum conditioning size in order to significantly increase the reliability of the statistical independence tests. Note that other similar 'Parent and Children' learning procedures were proposed recently in the machine learning literature, namely MMPC [10] and GetPC [3]. They could be used as well. Nonetheless HPC was favored in a recent evaluation using the same conditional independence test, over a range of different networks, sample sizes and number of variables [5, 7].

Formally, HPC can be viewed as an ensemble method for combining many weak PC learners in an attempt to produce a stronger PC learner. The algorithm was designed in order to endow the search procedure with the ability to: 1) handle efficiently data sets with thousands of variables but comparably few instances, 2) deal with datasets which present some deterministic relationships among the variables, 3) be correct under the faithfulness condition, and 4) be able to learn large neighborhoods. HPC is based on three subroutines: *Data-Efficient Parents and Children Superset* (DE-PCS), *Data-Efficient Spouses Superset* (DE-SPS), and *Interleaved Incremental Association Parents and Children* (Inter-IAPC), a weak PC learner based on Inter-IAMB [9] that requires little computation. HPC may be thought of as a way to compensate for the large number of false negative nodes, at the output of the weak PC learner with few data cases, by performing extra computations. HPC receives a target node T, a data set $\mathcal{D}$ and a set of variables $\mathbf{U}$ as input and returns an estimation of $\mathbf{PC}_T$. It is hybrid in that it combines the benefits of incremental and divide-and-conquer methods. The procedure starts by extracting a superset $\mathbf{PCS}_T$ of $\mathbf{PC}_T$ (line 1) and a superset $\mathbf{SPS}_T$ of $\mathbf{SP}_T$ (line 2) with a severe restriction on the maximum conditioning size ($\mathbf{Z} <= 2$) in order to significantly increase the reliability of the tests. A first candidate PC set is then obtained by running the weak PC learner on $\mathbf{PCS}_T \cup \mathbf{SPS}_T$ (line 3). The key idea is the decentralized search at lines 4-8 that includes, in the candidate PC set, all variables in the superset $\mathbf{PCS}_T \cup \mathbf{SPS}_T$ that have T in their vicinity. Note that, in theory, X is in the output of Inter-IAPC(Y) if and only if Y is in the output of Inter-IAPC(X). However, in practice, this may not always be true due the statistical test errors, especially with few data samples. The decentralized search enables the algorithm to handle large neighborhoods while still being correct under faithfulness condition. The correctness of is provided in Appendix A.

The essential graph is obtained by running HPC on the every node and by directing the *complelled* edges as shown in Algorithm 1. Note that HPC

Algorithm 1 *Recursive HPC*

Require: $\mathcal{D}$: data set; $\mathbf{U}$: the set of variables

Ensure: A PDAG $\mathcal{G}$ reprentative of the equivalence class containing the compelled edges

for all pair of nodes $X, Y \in \mathbf{U}$ **do**
    Create the link X-Y in $\mathcal{G}$ if $X \in HPC(Y)$ OR $Y \in HPC(X)$
end for
for all uncoupled meeting $X - Z - Y \in \mathcal{G}$ **do**
    if $Z \notin dSep(X, Y)$ **then**
        Orient $X - Z - Y$ as $X \rightarrow Z \leftarrow Y$
    end if
end for
repeat
    for all uncoupled meeting $X \rightarrow Z - Y$ **do**
        Orient $Z - Y$ as $Z \rightarrow Y$
    end for
    for all link $X - Y$ such that there is a path from X to Y **do**
        Orient $X - Y$ as $X \rightarrow Y$
    end for
    for all uncoupled meeting $X - Z - Y$ such that $X \rightarrow W$, $Y \rightarrow W$ and $Z - W$ **do**
        Orient $Z - W$ as $Z \rightarrow W$
    end for
until no more edges can be oriented

Algorithm 2 *HPC*

Require: T: target; $\mathcal{D}$: data set; $\mathbf{U}$: the set of variables

Ensure: $\mathbf{PC}_T$: Parents and Children of T

$[\mathbf{PCS}_T, \mathbf{dSep}] \leftarrow$ *DE-PCS*$(T, \mathcal{D})$
$\mathbf{SPS}_T \leftarrow$ *DE-SPS*$(T, \mathcal{D}, \mathbf{PCS}_T, \mathbf{dSep})$
$\mathbf{PC}_T \leftarrow$ *Inter-IAPC*$(T, \mathcal{D}(T \cup \mathbf{PCS}_T \cup \mathbf{SPS}_T))$
for all $X \in \mathbf{PCS}_T \setminus \mathbf{PC}_T$ **do**
    if $T \in$ *Inter-IAPC*$(X, \mathcal{D}(T \cup \mathbf{PCS}_T \cup \mathbf{SPS}_T))$ **then**
        $\mathbf{PC}_T \leftarrow \mathbf{PC}_T \cup X$
    end if
end for

Algorithm 3 *Inter-IAPC*

Require: T: target; D: data set; $\mathbf{U}$: set of variables;
Ensure: $\mathbf{PC}_T$: Parents and children of T;

$\mathbf{MB}_T \leftarrow \emptyset$
repeat
    ** Add true positives to* $\mathbf{MB}_T$
    $Y \leftarrow \mathbf{argmax}_{X \in (\mathbf{U} \setminus \mathbf{MB}_T \setminus T)} dep(T, X|\mathbf{MB}_T)$
    if $T \not\perp Y|\mathbf{MB}_T$ **then**
        $\mathbf{MB}_T \leftarrow \mathbf{MB}_T \cup Y$
    end if

    ** Remove false positives from* $\mathbf{MB}_T$
    for all $X \in \mathbf{MB}_T$ **do**
        if $T \perp X|(\mathbf{MB}_T \setminus X)$ **then**
            $\mathbf{MB}_T \leftarrow \mathbf{MB}_T \setminus X$
        end if
    end for
until $\mathbf{MB}_T$ has not changed

** Remove spouses of T from* $\mathbf{MB}_T$
$\mathbf{PC}_T \leftarrow \mathbf{MB}_T$
for all $X \in \mathbf{MB}_T$ **do**
    if $\exists \mathbf{Z} \subseteq (\mathbf{MB}_T \setminus X)$ such that $T \perp X \mid \mathbf{Z}$ **then**
        $\mathbf{PC}_T \leftarrow \mathbf{PC}_T \setminus X$
    end if
end for

Algorithm 4 *DE-PCS*

Require: T: target; $\mathcal{D}$: data set; $\mathbf{U}$: set of variables;
Ensure: $\mathbf{PCS}_T$: parents and children superset of T; **dSep**: d-separating sets;

Phase I: *Remove X if $T \perp X$*
$\mathbf{PCS}_T \leftarrow \mathbf{U} \setminus T$
for all $X \in \mathbf{PCS}_T$ **do**
    if $(T \perp X)$ **then**
        $\mathbf{PCS}_T \leftarrow \mathbf{PCS}_T \setminus X$
        $\mathbf{dSep}(X) \leftarrow \emptyset$
    end if
end for

Phase II: *Remove X if $T \perp X|Y$*
for all $X \in \mathbf{PCS}_T$ **do**
    for all $Y \in \mathbf{PCS}_T \setminus X$ **do**
        if $(T \perp X \mid Y)$ **then**
            $\mathbf{PCS}_T \leftarrow \mathbf{PCS}_T \setminus X$
            $\mathbf{dSep}(X) \leftarrow Y$
            break loop FOR
        end if
    end for
end for

Algorithm 5 *DE-SPS*

Require: T: target; $\mathcal{D}$: data set; $\mathbf{U}$: the set of variables; $\mathbf{PCS}_T$: parents and children superset of T; $\mathbf{dSep}$: d-separating sets;

Ensure: $\mathbf{SPS}_T$: Superset of the spouses of T;

$\mathbf{SPS}_T \leftarrow \emptyset$
for all $X \in \mathbf{PCS}_T$ **do**
    $\mathbf{SPS}_T^X \leftarrow \emptyset$
    for all $Y \in \mathbf{U} \setminus \{T \cup \mathbf{PCS}_T\}$ **do**
        if $(T \not\perp Y | \mathbf{dSep}(Y) \cup X)$ **then**
            $\mathbf{SPS}_T^X \leftarrow \mathbf{SPS}_T^X \cup Y$
        end if
    end for
    for all $Y \in \mathbf{SPS}_T^X$ **do**
        for all $Z \in \mathbf{SPS}_T^X \setminus Y$ **do**
            if $(T \perp Y | X \cup Z)$ **then**
                $\mathbf{SPS}_T^X \leftarrow \mathbf{SPS}_T^X \setminus Y$
                break loop FOR
            end if
        end for
    end for
    $\mathbf{SPS}_T \leftarrow \mathbf{SPS}_T \cup \mathbf{SPS}_T^X$
end for

must have found $dSep(X, Y)$ (at line 5 of RHPC) and have cached it for later retrieval. Aternatively, HPC can be run recursively on the adjacent nodes of a target variable in order to establish a local graph without having to construct the whole BN first as discussed in [4]. RHPC applies standard techniques at lines 4-19 to identify the compelled edges. The reader is directed to [2], pp. 538, for further details. The *correctness* and *completeness* of the edge orientation in RHPC are demonstrated in [1]. The correctness of RHPC follows from the correctness of HPC.

We now discuss the subroutines in more detail. Inter-IAPC (Algorithm 3) is a fast incremental method that receives a data set $\mathcal{D}$ and a target node T as its input and promptly returns a rough estimation of $\mathbf{PC}_T$, hence the term "weak" PC learner. Inter-IAPC is an straightforward extension of the algorithm Inter-IAMB [9]. Notice that neither MMPC [10] nor GetPC [3] should be used to implement this weak PC learner. The reason is that any break of symmetry of the PC relation in the output of these algorithm is an indication of a false positive member; the decentralized search would not aid in reducing the number of false positives variables at all. Inter-IAPC starts with a two-phase approach to infer $\mathbf{MB}_T$, that is, the Markov boundary of T. A growing phase attempts to iteratively add the best candidate variables to $\mathbf{MB}_T$, followed by a shrinking phase that attempts to remove as many irrelevant variables as possible, that is, the false positives in the current set $\mathbf{MB}_T$. The function $dep(T, X|\mathbf{MB}_T)$ at line 4 returns a statistical estimation of the association between T and X given the current set $\mathbf{MB}_T$. The shrinking phase is interleaved with the growing phase. Interleaving the two phases allows to eliminate as soon as possible some of the false positives in the current Markov blanket as the algorithm progresses during the Markov boundary search. $\mathbf{PC}_T$ is obtained by removing the spouses

of the target from the final $\mathbf{MB}_T$ (lines 14-19). Inter-IAPC is very fast and sound (the proof of soundness is provided in A), despite its *data-inefficiency* in practice. The decentralized search in HPC is an attempt to alleviate this problem as discussed earlier.

The subroutines DE-PCS (Algorithm 4) and DE-SPS (Algorithm 5) search a superset of $\mathbf{PC}_T$ and $\mathbf{SP}_T$ respectively with a severe restriction on the maximum conditioning size ($|\mathbf{Z}| <= 1$ in DE-PCS and $|\mathbf{Z}| <= 2$ in DE-SPS) in order to significantly increase the reliability of the tests. The variable filtering has two advantages : i) it allows HPC to scale to hundreds of thousands of variables by restricting the search to a subset of relevant variables, and ii) it eliminates many ADRs that produce many false negative errors in the ouput of the algorithm, as explained in the last section. DE-SPS works in two steps. First, a growing phase (lines 4-8) adds the variables that are d-separated from the target but still remain associated with the target when conditioned on another variable from $\mathbf{PCS}_T$. The shrinking phase (lines 9-16) discards irrelevant variables that are ancestors or descendants of a target's spouse. Pruning such irrelevant variables speeds up HPC.

References

[1] C. Meek. Causal inference and causal explanation with background knowledge. In *Proceedings of Eleventh Conference on Uncertainty in Artificial Intelligence*, pages 403–418. Morgan Kaufmann, August 1995.

[2] R. E. Neapolitan. *Learning Bayesian Networks.* Pearson Prentice Hall, Upper Saddle River, NJ, 2004.

[3] J. Peña, R. Nilsson, J. Björkegren, and J. Tegnér. Towards scalable and data efficient learning of Markov boundaries. *International Journal of Approximate Reasoning*, 45(2):211–232, 2007.

[4] J. M. Peña, J. Björkegren, and J. Tegnér. Growing Bayesian network models of gene networks from seed genes. *Bioinformatics*, 40:224–229, 2005.

[5] S. Rodrigues de Morais and A. Aussem. A novel scalable and data efficient feature subset selection algorithm. In *European Conference on Machine Learning and Principles and Practice of Knowledge Discovery in Databases, ECML-PKDD'08*, volume 5212 of *Lecture Notes in Computer Science*, pages 298–312, Antwerp, Belgium, 2008. Springer-Verlag Berlin Heidelberg.

[6] S. Rodrigues de Morais and A. Aussem. An efficient and scalable algorithm for local bayesian network structure discovery. In *European Conference on Machine Learning and Principles and Practice of Knowledge Discovery in Databases, ECML-PKDD'10*, Lecture Notes in Computer Science, Barcelona, Spain, 2010. Springer-Verlag Berlin Heidelberg, to appear.

[7] S. Rodrigues de Morais and A. Aussem. A novel Markov boundary based feature subset selection algorithm. *Neurocomputing*, 73:578–584, 2010.

[8] S. Rodrigues de Morais, A. Aussem, and M. Corbex. Handling almost-deterministic relationships in constraint-based Bayesian network discovery

: Application to cancer risk factor identification. In *16th European Symposium on Artificial Neural Networks ESANN'08*, pages 101–106, 2008.

[9] I. Tsamardinos, C. Aliferis, and A. Statnikov. Algorithms for large scale Markov blanket discovery. In *Florida Artificial Intelligence Research Society Conference FLAIRS'03*, pages 376–381, 2003.

[10] I. Tsamardinos, L. Brown, and C. Aliferis. The max-min hill-climbing Bayesian network structure learning algorithm. *Machine Learning*, 65(1):31–78, 2006.

A Proof of correctness

A structure learning algorithm from data is said to be correct (or sound) if it returns the correct DAG pattern (or a DAG in the correct equivalence class) under the assumptions that the independence tests are reliable and that the learning data set is a sample from a distribution P faithful to a DAG $\mathcal{G}$. The (ideal) assumption that the independence tests are reliable means that they decide (in)dependence if and only if the (in)dependence holds in P. Consequently, a parents and children learning algorithm is said to be correct (or sound) when under the assumptions that the independence tests are reliable and that the learning data set is a sample from a distribution P faithful to a DAG $\mathcal{G}$, the algorithm returns the correct set of parents and children of the target, that is, the target direct neighborhood. Correctness is a desirable asymptotic property though the underlying assumptions may not hold in practice. In general, we would want an edge to mean a direct dependence. Several definitions and intermediate theorems are required before we demonstrate *HPC*'s correctness under faithfulness condition.

Definition 1 *A Markov blanket $\boldsymbol{M}_T$ of T is any set of variables such that T is conditionally independent of all the remaining variables given $\boldsymbol{M}_T$. A Markov boundary, $\boldsymbol{MB}_T$, of T is any Markov blanket such that none of its proper subsets is a Markov blanket of T.*

Theorem 1 *Suppose $< \mathcal{G}, P >$ satisfies the faithfulness condition. Then for each variable X, the set of parents, children of X, and parents of children (spouses) of X is its unique Markov boundary.*

A proof can be found in [2]. Indeed, as $\mathbf{PCS}_T \cup \mathbf{SPS}_T$ is a subset of $\mathbf{U}$, a difficulty arises: a marginal distribution $P^{\mathbf{V}}$ of $\mathbf{V} \subset \mathbf{U}$ may not satisfy the faithfulness condition with any DAG even if $P^{\mathbf{U}}$ does. This is an example of embedded faithfulness, which is defined as follow:

Definition 2 *Let $P^{\boldsymbol{V}}$ be a distribution of the variables in $\boldsymbol{V}$ where $\boldsymbol{V} \subset \boldsymbol{U}$ and let $\mathcal{G} =< \mathbf{U}, \mathbf{E} >$ be a DAG. $< \mathcal{G}, P^{\boldsymbol{V}} >$ satisfies the embedded faithfulness condition if $\mathcal{G}$ entails all and only the conditional independencies in $P^{\boldsymbol{V}}$, for subsets including only elements of $\boldsymbol{V}$.*

We obtain embedded faithfulness by taking the marginal of a faithful distribution as shown by the next theorem:

Theorem 2 *Let $P^{\boldsymbol{U}}$ be a joint probability of the variables in $\boldsymbol{U}$ with $\boldsymbol{V} \subseteq \boldsymbol{U}$ and $\mathcal{G} =< \mathbf{U}, \mathbf{E} >$. If $< \mathcal{G}, P^{\boldsymbol{U}} >$ satisfies the faithfulness condition and $P^{\boldsymbol{V}}$ is the marginal distribution of $\mathbf{V}$, then $< \mathcal{G}, P^{\boldsymbol{V}} >$ satisfies the* embedded *faithful condition.*

The proof can be found in [2]. Note that not every distribution does admit an embedded faithful representation. This property is useful to prove the correctness of *HPC* under the faithfulness condition. Let $\mathbf{PC}_X^{\mathbf{U}}$ denote the variables $Y \in \mathbf{U}$ so that there is no set $\mathbf{Z} \subseteq \mathbf{U} \setminus \{X, Y\}$ such that $X \perp_P Y|\mathbf{Z}$. If $< \mathcal{G}, P^{\mathbf{U}} >$ satisfies the faithfulness condition, $\mathbf{PC}_X^{\mathbf{U}}$ are the parents and children of X in $\mathbf{U}$. In any case, $\mathbf{PC}_X^{\mathbf{U}}$ is the unique set of the variables Y_i that remain dependent on X conditioned on any set $\mathbf{Z} \subseteq \mathbf{U} \setminus \{X, Y_i\}$.

Theorem 3 *Let $\boldsymbol{U}$ be a set of random variables and $\mathcal{G} =< \mathbf{U}, \mathbf{E} >$ be a DAG. If $< \mathcal{G}, P^{\boldsymbol{U}} >$ satisfies the faithfulness condition, then every target T admits a unique Markov boundary $\boldsymbol{MB}_T^{\boldsymbol{U}}$. Moreover, for all $\boldsymbol{V}$ such that $\boldsymbol{MB}_T^{\boldsymbol{U}} \subseteq \boldsymbol{V} \subseteq \boldsymbol{U}$, T admits a unique Markov boundary over $\boldsymbol{V}$ and $\boldsymbol{MB}_T^{\boldsymbol{V}} = \boldsymbol{MB}_T^{\boldsymbol{U}}$.*

Proof: If $\mathbf{MB}_T^{\mathbf{U}}$ is the Markov boundary of T in $\mathbf{U}$, then T is independent of all variable $Y \in [\mathbf{V} \setminus (\mathbf{MB}_T^{\mathbf{U}} \cup T)]$ conditionally on $\mathbf{MB}_T^{\mathbf{U}}$, then $\mathbf{MB}_T^{\mathbf{U}}$ is a Markov blanket in $\mathbf{V}$. Moreover, none of the proper subsets of $\mathbf{MB}_T^{\mathbf{U}}$ is a Markov blanket of T in $\mathbf{V}$, so $\mathbf{MB}_T^{\mathbf{U}}$ is also a Markov boundary of T in $\mathbf{V}$. So if it is not the unique MB for T in $\mathbf{V}$ there exists some other set $\mathbf{S}_T$ not equal to $\mathbf{MB}_T^{\mathbf{U}}$, which is a MB of T in $\mathbf{V}$. Since $\mathbf{MB}_T^{\mathbf{U}} \neq \mathbf{S}_T$ and $\mathbf{MB}_T^{\mathbf{U}}$ cannot be a subset of $\mathbf{S}_T$, there is some $X \in \mathbf{MB}_T^{\mathbf{U}}$ such that $X \notin \mathbf{S}_T$. Since $\mathbf{S}_T$ is a MB for T, we would have $T \perp_P X|\mathbf{S}_T$. If X is a parent or child of T, we would not have $T \perp_{\mathcal{G}} X|\mathbf{S}_T$ which means we would have a conditional independence that is not entailed by d-separation in $\mathcal{G}$, which contradicts the faithfulness condition. If X is a parent of a child of T in $\mathcal{G}$, let Y be their common child in $\mathbf{U}$. If $Y \in \mathbf{S}_T$ we again would not have $T \perp_{\mathcal{G}} X|\mathbf{S}_T$. If $Y \notin \mathbf{S}_T$ we would have $T \perp_P Y|\mathbf{S}_T$ because $\mathbf{S}_T$ is a MB of T in $\mathbf{V}$ but we do not have $T \perp_{\mathcal{G}} Y|\mathbf{S}_T$ because T is a parent of Y in G. So again we would have a conditional independence which is not a d-separation in $\mathcal{G}$. This proves that there can not be such set $\mathbf{S}_T$. $\square$

Theorem 4 *Let $\boldsymbol{U}$ be a set of random variables and T a target variable. Let $\mathcal{G} =< \mathbf{U}, \mathbf{E} >$ be a DAG such that $< \mathcal{G}, P^{\boldsymbol{U}} >$ satisfies the faithfulness condition. Let $\boldsymbol{V}$ be such that $\boldsymbol{MB}_T^{\boldsymbol{U}} \subseteq \mathbf{V} \subseteq \boldsymbol{U}$ then, $\boldsymbol{PC}_T^{\boldsymbol{V}} = \boldsymbol{PC}_T^{\boldsymbol{U}}$.*

Proof: Clearly $\mathbf{PC}_T^{\mathbf{U}} \subseteq \mathbf{PC}_T^{\mathbf{V}}$ as $\mathbf{MB}_T^{\mathbf{U}} \subseteq \mathbf{V} \subseteq \mathbf{U}$. If $X \in \mathbf{PC}_T^{\mathbf{V}}$ and $X \notin \mathbf{PC}_T^{\mathbf{U}}$, $\exists \mathbf{Z} \subseteq \mathbf{MB}_T^{\mathbf{U}} \setminus X$ such that $T \perp_P X|\mathbf{Z}$ because all non adjacent nodes may be d-separated in $\mathcal{G}$ by a subset of its Markov boundary. As $\mathbf{MB}_T^{\mathbf{U}} = \mathbf{MB}_T^{\mathbf{V}}$ owing to Theorem 3, so X and T can be d-separated in $\mathbf{V} \setminus \{X, T\}$. Therefore, X cannot be adjacent to T in $\mathbf{V}$. $\square$

Theorem 5 *Let $\boldsymbol{U}$ be a set of random variables and T a target variable. Let $\mathcal{G} =< \mathbf{U}, \mathbf{E} >$ be a DAG such that $< \mathcal{G}, P^{\boldsymbol{U}} >$ satisfies the faithfulness condition. Let $\boldsymbol{V}$ be such that $\boldsymbol{MB}_T^{\boldsymbol{U}} \subseteq \boldsymbol{V} \subseteq \boldsymbol{U}$. Under the assumption that the independence tests are reliable, Inter-IAPC$(T, \mathcal{D}, \boldsymbol{V})$ returns $\boldsymbol{PC}_T^{\boldsymbol{U}}$. Moreover, let $X \in \boldsymbol{V} \setminus T$, then $\forall T \in \boldsymbol{V}$, T is in the output of Inter-IAPC$(X, \mathcal{D}, \boldsymbol{V})$ iff $X \in \boldsymbol{PC}_T^{\boldsymbol{U}}$.*

Proof: We prove first that *Inter-IAPC*$(T, \mathcal{D}, \mathbf{V})$ returns $\mathbf{PC}_T^{\mathbf{U}}$. In lines 1-13, *Inter-IAPC* seeks a minimal set $\mathbf{S}_T \subseteq \mathbf{V} \setminus T$ that renders $\mathbf{V} \setminus \mathbf{S}_T$ independent of T conditionally on $\mathbf{S}_T$. This set is unique owing to Theorem 3, therefore $\mathbf{S}_T = \mathbf{MB}_T^{\mathbf{V}} = \mathbf{MB}_T^{\mathbf{U}}$. In the backward phase, *Inter-IAPC* removes the variables $X \in \mathbf{MB}_T^{\mathbf{V}}$ such that $\exists \mathbf{Z} \subseteq (\mathbf{MB}_T^{\mathbf{V}} \setminus X)$ for which $T \perp X \mid \mathbf{Z}$. These variables are the spouses of T in $\mathcal{G}$, so *Inter-IAPC*$(T, \mathcal{D}, \mathbf{V})$ returns $\mathbf{PC}_T^{\mathbf{U}}$. Now, if $X \notin \mathbf{PC}_T^{\mathbf{U}}$ then $X \notin \mathbf{PC}_T^{\mathbf{V}}$ owing to Theorem 4. So there is a set $\mathbf{Z} \subseteq \mathbf{V} \setminus \{X, Y\}$ such that $T \perp X \mid \mathbf{Z}$. Therefore, X cannot be in the output of *Inter-IAPC*$(T, \mathcal{D}, \mathbf{V})$, nor T can be in the output of *Inter-IAPC*$(X, \mathcal{D}, \mathbf{V})$. $\square$

Theorem 6 *Under the assumptions that the independence tests are reliable and that the data set is a sample from a probability distribution $P^{\mathbf{U}}$ faithful to a DAG $\mathcal{G}$, then $HPC(T, \mathcal{D}, \mathbf{U})$ returns $\mathbf{PC}_T^{\mathbf{U}}$.*

Proof: Let $\mathbf{V} = (\mathbf{PCS} \cup \mathbf{SPS})$, then $\mathbf{V}$ is a superset of $\mathbf{MB}_T^{\mathbf{U}}$. Based on what is stated by Theorem 3 we know that $\mathbf{MB}_T^{\mathbf{V}} = \mathbf{MB}_T^{\mathbf{U}}$. If T is in the output of *Inter-IAPC*$(X, \mathbf{V}, \mathcal{D})$ then X should be in the output of *Inter-IAPC*$(T, \mathbf{V}, \mathcal{D})$ owing to Theorem 5. So *HPC* returns $\mathbf{PC}_T^{\mathbf{U}}$. $\square$.
